# Neutrophil extracellular traps promote lung colonization of triple-negative breast cancer by inducing mesenchymal to epithelial transition

**DOI:** 10.3389/fimmu.2026.1892317

**Published:** 2026-09-16

**Authors:** Taiko Kawakami, Yusuke Nakanishi, Akari Suzuki, Kazuhiko Yamamoto, Kyoko Takahashi

**Affiliations:** 1 College of Bioresource Sciences, Graduate School of Bioresource Sciences, Nihon University, Fujisawa, Kanagawa, Japan; 2 Laboratory for Autoimmune Diseases, RIKEN Center for Integrative Medical Sciences, Yokohama, Kanagawa, Japan

**Keywords:** mesenchymal epithelial transition (MET), metastasis, neutrophil extracellular traps (NETs), TGF-*β* signaling, tumor microenvironment

## Abstract

Metastatic recurrence is still a major problem for triple-negative breast cancer (TNBC). Neutrophil extracellular traps (NETs) in the pulmonary microenvironment are known to play key roles in the metastatic colonization and outgrowth of TNBC. However, the exact role that NETs play in determining the phenotypic plasticity and reactivation of disseminated tumor cells (DTCs) during acute relapse remains unclear. Here, we utilized an orthotopic, acute recurrent metastasis mouse model by transplanting 4T1 cells into wild-type or NET-deficient (*Padi4^-/-^*) mice, followed by primary tumor resection. Although *Padi4* deficiency had no effect on the growth of the primary tumors, it significantly reduced lung metastasis and prolonged survival. 4T1 cells re-established from WT lungs displayed a highly proliferative epithelial phenotype, whereas those from *Padi4^-/-^* mice appeared to maintain a quiescent mesenchymal state. Crucially, tissue-adapted NET-forming pulmonary neutrophils could facilitate a mesenchymal-to-epithelial transition (MET)-like switch by robustly upregulating transforming growth factor-beta (TGF-*β*) negative regulators, thereby attenuating autocrine signaling. Collectively, our findings suggest that tissue-adapted NETs serve as crucial microenvironmental cues that support the exit from quiescence and drive metastatic outgrowth, highlighting a promising perioperative therapeutic target for TNBC.

## 1 Introduction

When cancer spreads from its primary site to a distant part of the body, it is called metastatic cancer. Metastasis is the major cause of cancer mortality, accounting for about 90% of cancer-related deaths (1, 2). Metastasis often results from relapses or recurrence, where metastases manifest following definitive treatment such as local surgery and/or systemic therapy. In case of breast cancer, the second most common cancer among women worldwide (3), metastatic recurrence is a major problem. Triple-negative breast cancer (TNBC) has a poor prognosis due to its aggressive form, high recurrence risk, and lack of specific targets (4). Unlike other solid tumors, breast cancer is characterized by a wide window of metastatic recurrence, spanning months to decades after diagnosis. However, TNBC and HER2-positive breast cancers are associated with a shorter latency period to metastatic relapse, while Luminal-type breast cancers exhibit a prolonged time to recurrence, often linked to the quiescence (dormancy) of metastasized tumor cells (5, 6).

Metastasis formation initiates when a single or cluster of cancer cells detaches from the primary tumors and subsequently invades the bloodstream. These circulating tumor cells (CTCs) eventually extravasate from blood vessels and disseminate to distant organs such as the lungs, liver, and bone marrow, where they are termed disseminated tumor cells (DTCs). Among these sites, the lungs are the most frequent organ for early metastatic relapses in TNBC patients (5). This high incidence of lung metastasis underscores the clinical urgency of elucidating the mechanisms governing the establishment and outgrowth of DTCs within the pulmonary microenvironment. Phenotypical changes along the epithelial-mesenchymal transition (EMT) pathway are thought to critically control CTCs and DTCs metastatic potential (7, 8). Liu et al., demonstrated in a breast cancer mouse model that while mesenchymal CTCs possess only limited metastatic potential, epithelial-like cells exhibiting restricted mesenchymal transition exhibit the strongest metastatic formation capacity (9). This finding highlights the importance of epithelial plasticity in metastatic success. However, the precise mechanisms by which these DTCs interact with the metastatic microenvironment to acquire the ability to form overt metastatic foci remain poorly understood.

The tumor microenvironment (TME) is a dynamic and complex ecosystem composed of cancer cells and diverse non-cancer cell types, including immune cells such as macrophages and neutrophils, fibroblasts, and endothelial cells. Accumulating evidence in recent years has led to the recognition that these non-malignant host cells actively participate in tumor progression, particularly during the metastatic process (10). Neutrophils are one of the most abundant immune cell types in TME, and it has been evidenced that the structures released by neutrophils, known as neutrophil extracellular traps (NETs), play a critical role in breast cancer metastasis. In a breast cancer mouse model, NETs protect CTCs and promote their adhesion to the vasculature via the formation of a pre-metastatic niche – an environment suitable for cancer cell engraftment in the lung metastasis (11). In addition, Adrover et al., demonstrated that intravascular aggregates of neutrophils and NETs caused occlusion of the tumor vasculature, leading to hypoxia and necrosis, which in turn promoted lung metastases (12). Furthermore, we and others previously reported that NETs affect primary tumor development through direct cytotoxicity or interfering with effector anti-tumor response by T cells and NK cells (13, 14). Thus, while the role of NETs in various steps of cancer progression, including primary tumor dynamics and pre-metastatic niche preparation, has been increasingly recognized, their specific role as a direct ‘metastatic niche’- specifically, the process by which cancer cells arrive at the metastatic site and establish metastatic foci there- remains to be fully elucidated.

In this study, we investigated the role of NETs in the process of TNBC metastasizing the lungs using an acute recurrent metastasis mouse model that mimics clinical symptoms and revealed that NETs play a vital role in TNBC outgrowth. Furthermore, we examined how NETs promote the reactivation of TNBC, suggesting that they may acquire proliferative capacity by inducing mesenchymal epithelial transition (MET)-like behavior through TGF-*β* signaling blockade.

## 2 Materials and methods

### 2.1 Mice

BALB/c mice were purchased from SLC Japan (Shizuoka, Japan). *Padi4*-deficient (*Padi4^-/-^*) mice, C57BL/6J background (B6) mice were kindly provided by Dr. A. Suzuki and Dr. K. Yamamoto and backcrossed with BALB/c in our facility as described previously (14). The PCR primer sequences used for genotyping were Padi4_F: CAGTGGGTCAGTGACTGTC, Padi4_R: CGAGAGCTAGCCTGGGATC, Neo_F: CAGCTGTGCTCGACGTTGTC, Neo_R: CAACGCTATGTCCTGATAGC. The mice were used at 6–8 weeks of age. All experiments were approved by the Nihon University Animal Care and Use Committee and conducted in accordance with their guidelines.

### 2.2 Cell culture

Murine 4T1 breast cancer cells were purchased from American Type Culture Collection (ATCC: Manassas, VA, USA). To evaluate the phenotypic differences of 4T1 cells within the pulmonary environment, 4T1 cells were re-isolated from the lungs of tumor-resected mice at 17 days after surgery. Entire lung lobes were harvested from both WT and *Padi4^-/-^* mice. The whole lung tissues were minced and enzymatically digested with 25 μg/mL Liberase™ (Roche, Basel, Switzerland) with vigorous agitation (160 rpm., 37 °C, 30 min). The resulting single-cell suspensions were washed and resuspended in growth medium. To selectively recover 4T1 cells from the lung cell mixture, cells were cultured in medium supplemented with 60 μM 6-thioguanine (6-TG) (Sigma-Aldrich, Saint Louis, USA). After 10 days of selection, the re-established cell line was designated as Lu-WT (Lung-derived from WT mice), Lu-KO (lung-derived from *Padi4^-/-^* mice). These cells were used for subsequent assays within 2 or 3 passages to minimize phenotype drift. All cell lines were cultured in complete media containing RPMI 1640 medium (Wako Chemicals Osaka, Japan) supplemented with 10% of FBS (Capricorn Scientific, Ebsdorfergrund, Germany), 1% of penicillin and streptomycin, and 5 µM 2-mercaptoethanol (Nacalai Tesque, Kyoto, Japan).

### 2.3 Mouse experiments

To generate the tumor-bearing mice, 4T1 cells (10^4^) were suspended in a mixture of PBS and 50% Matrigel (Corning, New York, USA) and injected subcutaneously into the 4^th^ mammary fat pad of female WT or *Padi4^-/^*^-^ mice. The width and length of the tumors were measured every 3 or 4 days with a caliper. The tumor volume was calculated using the following formula: ½ (width^2^ × length). The primary tumor as well as draining lymph nodes were surgically resected, and incisions closed with wound clips under 2% isoflurane inhalation once the tumor size reached 180–200 mm^2^ as previously described (15). For pain control, carprofen (5 mg/kg body weight) was administrated 2–3 hours before surgery and 24 hours after surgery. Mice were euthanized by cervical dislocation under deep anesthesia (3-5% isoflurane inhalation) at designated timepoints. When mice were unable to ambulate to reach food/water, or showed signs of respiratory disease due to the metastasis, they were euthanized, and the date was recorded for survival rate calculation.

### 2.4 Hematoxylin and eosin staining

Mouse lungs were perfused with PBS to remove blood and then fixed in 4% paraformaldehyde (PFA) overnight at 4°C. After washing three times, lungs were embedded in paraffin and sectioned at 6 μm. Sections were stained with hematoxylin and eosin (H&E) for histological evaluation. Images were obtained using BZ-X800 all-in-one fluorescence microscope (Keyence, Osaka Japan).

### 2.5 Immunofluorescence

For immunohistochemistry (IHC), after fixing 4% PFA overnight, lungs were immersed in 15% sucrose overnight and 30% sucrose for 2 days at 4°C Moisture was wiped off lungs and frozen in SCEM-L1 (SECTION-lab Inc, Hiroshima, Japan) at -80°C. Frozen lungs were cut into 6 μm sections using Cryostat. For immunocytochemistry (ICC), cells were seeded in Camber-slide (Matsunami Glass Osaka, Japan) and cultured in the presence of 10 μM SB431542 (TGF-*β* receptor kinase inhibitor) in certain experiment. To evaluate NET formation *ex-vivo*, pulmonary neutrophils were isolated from lung single cell-suspension via MACS using Neutrophil Isolation Kit (Miltenyi Biotech), then were seeded in Chamber-slide. They were then permeabilized by 0.1% TritonX-100 in PBS or acetone/methanol and blocked with 10% BSA/5% donkey serum in PBS. The slides were incubated with the corresponding primary antibodies (Citrullinated histone H3 (Cit-H3: Abcam), E-cadherin (Cell signaling technology: CST)) overnight at 4°C. The next day, they were washed three times in PBST and stained with the corresponding secondary antibodies for 1h at room temperature. Specimens were counterstained with DAPI (4′,6-diamidino-2-phenylindole) and mounted with antifade medium (ProLong Gold, Thermo Fisher Scientific, Waltham, MA, USA), observed with a TCS SP8 LIGHTNING microscope and images were acquired using LAS X software (Leica Microsystems, Wetzlar, Germany).

### 2.6 Flow cytometry analysis

Lung excised on the indicated day were minced with scissors and treated with 25 μg/ml Liberase ™ (Roche, Basel, Switzerland) with vigorous agitation (160 rpm., 37 °C, 30 min). The cell suspension was passed through a cell strainer (Corning, New York, USA) and treated with RBC lysis buffer (BioLegend, California, USA). After washing PBS containing 1% FBS and 1 μM EDTA, cells were stained with a combination of anti-CD11b, anti-Ly6C, anti-Ly6G, anti-CD3, anti-CD19, anti-NK1.1, anti-Ter119, anti-Siglec-F, anti-CD45 and 7AAD after blocking Fc receptors. Flow cytometry and neutrophil sorting were performed on FACSAria II. For EpCAM assays, cells were stained with anti-EpCAM antibody (BioLegend) after Fc block. The FlowJo software was used for data analysis.

### 2.7 RNA extraction and quantitative RT-PCR

Total RNA was extracted with FastGene RNA isolation kit (Nippon Genetics EUROPE) following the manufacturer’s instructions. Complementary DNA was synthesized by SuperScript IV Reverse Transcriptase (Thermo Fisher Scientific) and amplified using SYBR Green Master Mix (Kapa Biosystems). Target mRNA expression was normalized to that of *Gapdh*. The primer sequences used for qPCR are listed in **Supplementary Table**.

### 2.8 RNA-seq analysis of re-established cell line

Total RNA was extracted from Lu-WT cells or Lu-KO cells as described above (n = 3 representative samples selected for library preparation). RNA quality was assessed using Agilent TapeStation system. Libraries were prepared using the SMART-seq mRNA Kit according to the manufacturer’s protocol. After construction and validation, libraries were subjected to paired-end sequencing on the illumina NovaSeq platform. Sequence analysis was performed using RNA-SeqChef (16), with reads mapped to the mouse genome.

### 2.9 Western blotting analysis

Quantification of western blotting bands was performed as described previously (17). Primary antibody (anti Smad2/3) and secondary antibody (HRP-labeled anti Rabbit-IgG) were purchased from CST. Loading control was detected using HRP-conjugated anti β-actin antibody (Abcam).

### 2.10 Co-culture experiment

Sorted neutrophils were co-cultured with Lu-KO cells at a 1:1 ratio in RPMI medium containing 1% FBS for 48 hours. To remove neutrophils, the cells were washed three times with cold PBS. Adherent cells were directly analyzed for Immunohistochemistry or were harvested for qPCR. To degrade NET structures, recombinant DNase I (1.5 U/ml) was added to the co-culture medium.

### 2.11 Statistical analysis

Statistical analyses were performed using Prism 8 software (GraphPad, San Diego, CA, USA). A *t*-test with Welch’s correction was used for statistical analyses of two-group comparisons. Multigroup comparisons were performed using one-way analysis of variance (ANOVA) followed by Tukey’s multiple comparison test (*p < 0.05, **p < 0.01). Data are presented as individual values with the mean and 95% confidence intervals. For analysis of survival after tumors resection, each group was subjected to Kaplan-Meier analysis and comparisons were performed using the log-rank test.

## 3 Results

### 3.1 NETs are essential for lung metastasis in an acute recurrence model of TNBCs.

To address the role of NETs in lung metastasis of TNBCs, we subcutaneously transplanted 4T1 cells into the 4^th^ mammary fat pad of wild-type or *Padi4^-/-^* mice (peptidylarginine deiminase 4-deficient, lacking NET formation capability) (BALB/c background) respectively, and subsequently established tumor metastasis models by surgical removal of the primary tumor (**Figure 1A**). We previously reported that *Padi4^-/-^* significantly diminished tumor growth (14). However, in a model using orthotopic transplantation of 4T1 cells, no difference in tumor growth was observed (**Figure 1B**), unlike when the cells were transplanted ectopically into the flank. To elucidate the cellular mechanism underlying this observation, we characterized the immune cells infiltration within the primary orthotopic tumors using flow cytometry. The proportions of the major myeloid cell populations including monocytic myeloid – derived suppressor cells (mMDSCs), neutrophils, and macrophages, were comparable between WT and *Padi4^-/-^* mice (**Supplementary Figures 1A, B**). These results indicated that Padi4 deficiency does not alter the composition of these immune cell populations within the primary tumor microenvironment. Nevertheless, *Padi4^-/-^* mice significantly increased survival compared to the wild type mice after the primary tumor resections (**Figure 1C**). When the lungs were harvested 17 days after resection, only limited macroscopic metastatic lesions were observed in the majority of *Padi4^-/-^* mice (**Figure 1D**). These observations were consistent with histological analysis, which also revealed only limited metastatic lesions in the *Padi4^-/-^* mice (**Figure 1E**). Flow cytometric analysis demonstrated that no significant differences in the proportion of infiltrating neutrophils among total immune cells were observed between the two groups (**Figure 1F**). Notably, despite their equivalent proportions, immunocytochemistry of isolated pulmonary neutrophils showed that WT neutrophils produced abundant Cit-H3^+^ NETs, whereas Padi4^-/-^ neutrophils lacked Cit-H3^+^ staining (**Figure 1G**), indicating that *Padi4*-independent NET formation (18) was not observed in this metastasis model. Additionally, localized NETs within lung tissue sections were confirmed in WT metastatic lesions (**Supplementary Figure 2**). Collectively, these observations indicated that Padi4 deficiency impairs lung metastasis in association with the loss of NET formation, without causing a systemic depletion of pulmonary neutrophils.

**Figure 1:**
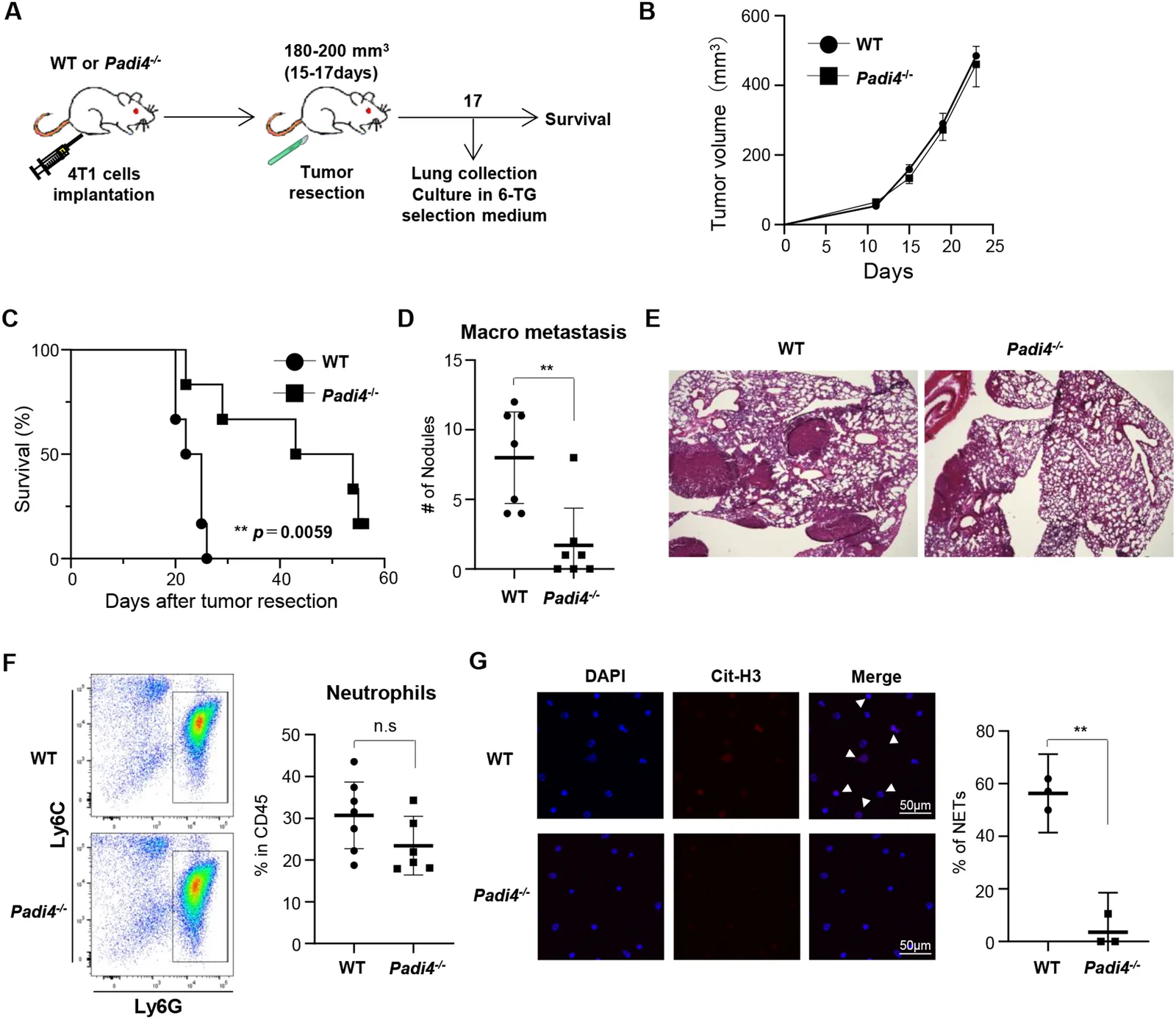
*Padi4* dependent NETs were required for the lung metastasis of TNBC. **(A)** Schematic diagram of experimental design. 4T1 cells were orthotopic transplanted into 4^th^ mammary fat pad of BALB/c WT or *Padi4^-/-^* mice. Primary tumors were subsequently surgical removed to mimic acute recurrence of TNBC. **(B)** Tumor volumes were measured at the indicated time points (n = 7). **(C)** Kaplan-Meier survival curve of WT mice and *Padi4^-/-^* mice (n = 6) following primary tumor resection. *p* values were determined using the log-rank test. **(D–G)** Lungs were taken for analysis 17 days after primary tumor resection. **(D)** The number of visible lung metastasis was counted under stereo microscope. **p < 0.01.; *t*-test with Welch’s correction. **(E)** H&E staining of lung tissue from WT (left) or *Padi4^-/-^* (right) mice. **(F)** Representative plot of infiltrating neutrophils (left) and percentage of those population within CD45^+^ cells. Data are presented as individual points with mean and 95% confidence intervals. n.s means not significant. **(G)** Representative immunocytochemistry images of isolated pulmonary neutrophils staining for DAPI (blue) and Cit-H3 (red), confirming NET formation (left) and percent of NET forming cells (right). Data are shown as individual data points (n = 3) with mean and 95% confidence intervals. The *p*-values were calculated using *t-*test with Welch’s correction. **p < 0.01.

### 3.2 NETs induce epithelial phenotypic transition

EMT is a typical process demonstrating cellular plasticity; it refers to the reversible ability of epithelial cells to dedifferentiate or transdifferentiate into a mesenchymal gene program, accompanied by morphological and phenotypic changes (19, 20). In recent years, it has been shown that epithelial-mesenchymal plasticity is associated with enhanced stem cell-like properties and metastatic potential. This plasticity is, in part, associated with the ability to reverse EMT through MET. While MET is a critical event in embryonic development, it is increasingly recognized as important in cancer metastasis. Once disseminated tumor cells reach distant organs, they revert to a more epithelial-like state and acquire proliferative capacity to promote metastatic colony formation and growth. Otherwise, an irreversible, extreme state of EMT would impair metastatic potential, as demonstrated by numerous metastasis studies in animals and more recent lineage tracing studies (21–23). Based on these reports, we hypothesized that NETs may trigger the colonization of TNBC cells during the lung metastasis by inducing MET-like transition. To test this hypothesis, lungs from WT or *Padi4^-/-^* mice were harvested 17 days after primary tumor resection (**Figure 1A**) for the recovery of 4T1 cells through selection with 6-TG (9). Significant differences in morphology were observed among the parent lines 4T1, Lu-WT (4T1 re-isolated from WT mice), and Lu-KO (4T1 re-isolated from *Padi4^-/-^* mice) (**Figure 2A**). Parental 4T1 cells and Lu-WT displayed a typical epithelial phenotype with tight cell-cell contact, whereas Lu-KO cells displayed a mesenchymal, spindle-shaped phenotype with loss of cell-cell adhesion. Consistent with morphological observations, the expression of classical epithelial markers such as EpCAM and E-cadherin (*Cdh1*) was higher in both the parental lines and Lu-WT at both the protein (**Figure 2B**) and the gene levels (**Figure 2C**), while the expression of the classical mesenchymal marker vimentin was higher in Lu-KO (**Figure 2C**). It has been reported that EpCAM^-^ and CD106^+^CD51^+^ cells within the hybrid/intermediate EMT state population were the most plastic and aggressive populations (19, 24). Interestingly, we found that CD106 expression was specifically downregulated only in Lu-WT cells, while parental 4T1 and Lu-KO cells maintained higher levels (**Figure 2C**). Regarding CD51, although its expression was lowest in the parental 4T1 line, it was significantly upregulated in Lu-KO cells. Notably, this upregulation was significantly attenuated in Lu-WT cells compared to Lu-KO cells. These results suggested that while dissemination to the pulmonary environment may initially trigger a shift toward a plastic hybrid state, the microenvironment of WT mice -where NET forming neutrophils are present- is associated with an exit from this hybrid state toward a more committed epithelial-like phenotype. To further confirm whether MET-like transition from hybrid EMT leads to the acquisition of proliferation capacity, we performed Ki-67 staining. Immunofluorescence analysis revealed that Lu-WT cells exhibited significantly higher proportion of Ki-67 positive cells as well as E-cadherin compared to Lu-KO cells (**Figures 2D, E**). Collectively, these findings highlight a strong link between neutrophil/NET-associated microenvironmental signals and the re-expression of epithelial traits during lung colonization.

**Figure 2:**
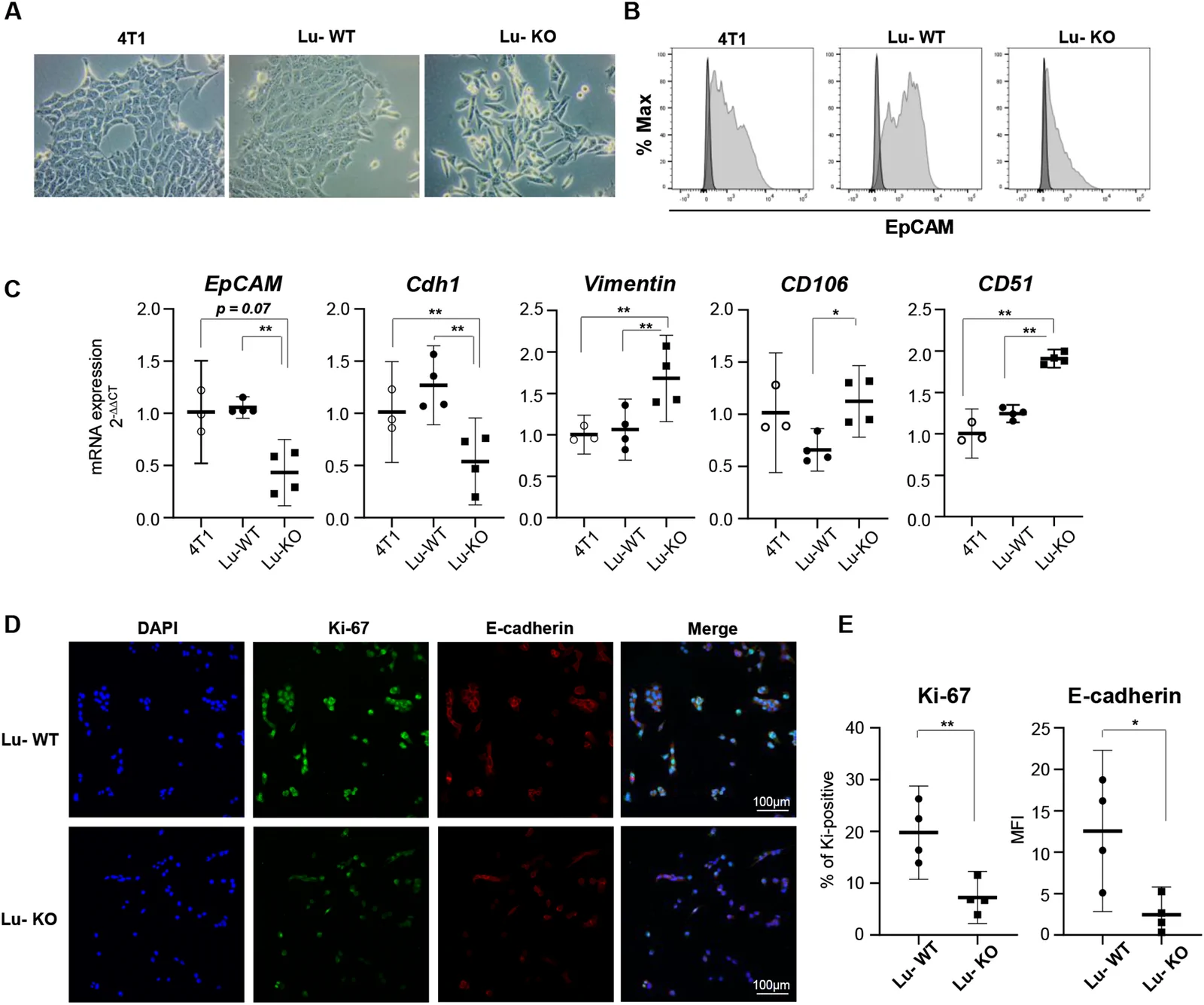
Phenotype of re-isolated cancer cells from lung in 4T1 metastasis mouse model. **(A)** Morphology of parental 4T1, 4T1 samples re-isolated from lung of wild type mice (Lu-WT) and samples re-isolated from lung of *Padi4^-/-^* mice (Lu-KO). Shown are representative pictures of each cell line. **(B)** EpCAM expression in 4T1, Lu-WT and Lu-KO was determined by flow cytometry. Representative histograms with EpCAM staining in gray and isotype controls in black. **(C)** mRNA transcript levels of indicated markers in 4T1, Lu-WT and Lu-KO were assessed upon quantitative RT-PCR with specific primer and *Gapdh* as a housekeeping gene. Data are shown as mean and 95% confidence intervals from n = 3–4 individual samples. **(D)** Representative immunofluorescence images of Ki-67 staining (green) and E-cadherin (red). **(E)** Percent of Ki-67 positive cells (left) and mean fluorescence intensity (MFI) of E-cadherin expression (right) were calculated using ImageJ software. Data are shown as individual data points (n = 4) with mean and 95% confidence intervals. The *p*-values were calculated using *t-*test with Welch’s correction or one-way ANOVA followed by Tukey’s multiple comparison test. *p < 0.05. **p < 0.01.

### 3.3 Identification of TGF-*β* signaling as a key mediator of the epithelial phenotypic transition

To elucidate the molecular mechanisms underlying the NET-induced epithelial switch, we conducted bulk RNA-sequencing and characterized the profiles of differentially expressed genes (DEGs) between Lu-WT and Lu-KO using the RNA-SeqChef pipeline. There were 380 genes that were up-regulated, and 271 genes that were down-regulated in Lu-WT (**Figure 3A**). Based on Gene Ontology (GO) cellular component analysis, the DEGs were enriched in categories related to cell-cell junction, apical junction complex, and tight junction, indicating that NETs trigger an MET-like epithelial shift (**Figure 3B**). Furthermore, analysis of the MsigDB hallmark gene set showed that the DEGs were enriched for TNFα signaling via NF-κB, unfolded protein response, hypoxia, TGF-*β* signaling and cholesterol homeostasis. Pathway interaction database (PID) analysis further identified enrichment of TGFβR signaling (**Figures 3C, D**). Consequently, we hypothesized that TGF-*β* signaling might be regulated by NETs. Notably, the genes that were consistently identified as negative regulators of TGF-*β* including *Smad7*, *Smurf1*, *Skil*, and *Ppp1r15a*- showed significantly increased expression in Lu-WT, which was subsequently confirmed by quantitative PCR (**Figure 3E**).

**Figure 3:**
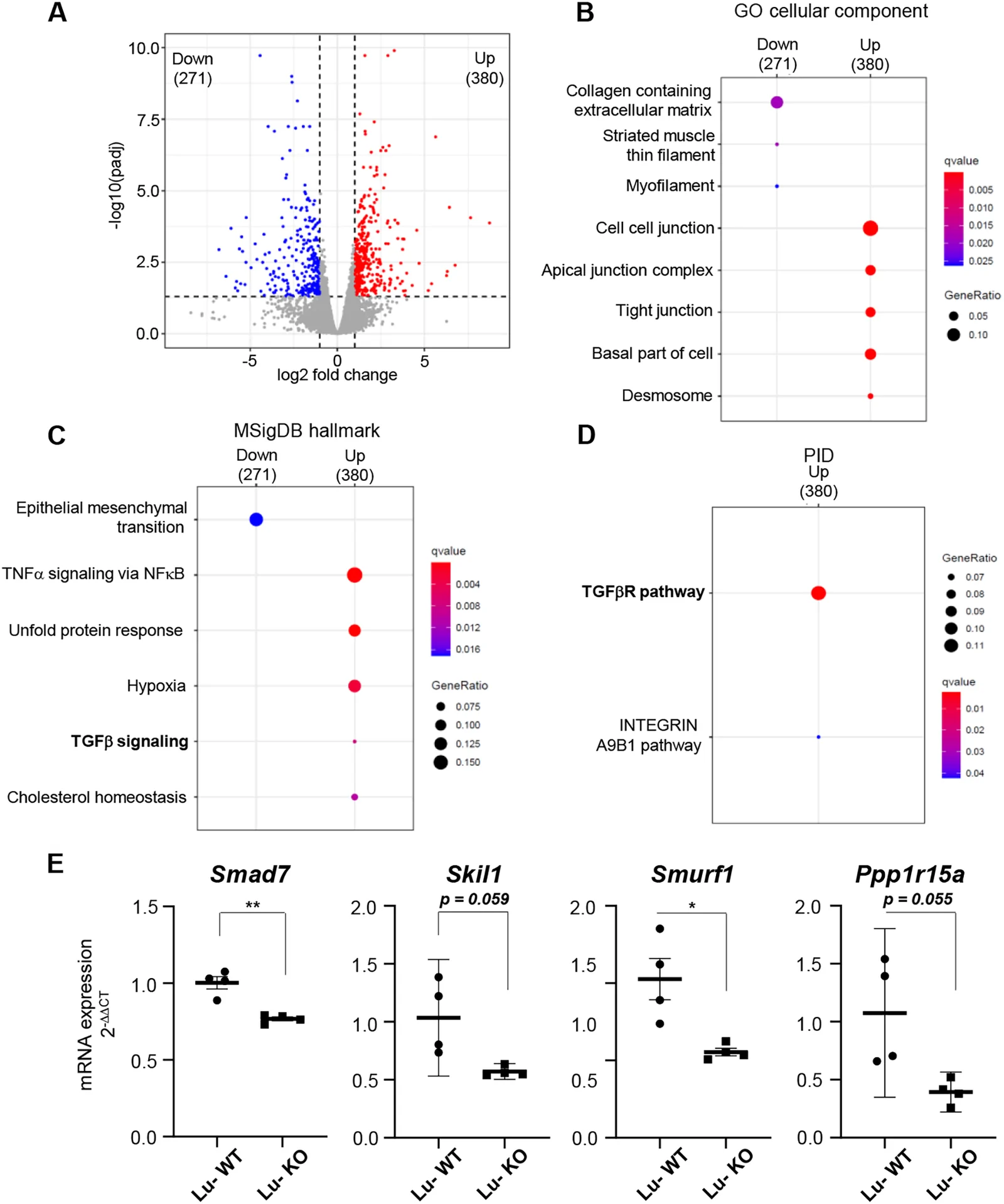
Bulk RNA-seq analysis and gene set enrichment analysis. **(A)** Volcano plot showing differential expression of genes. Three 4T1 samples re-isolated from lung of wild type mice (Lu-WT) and three samples re-isolated from lung of *Padi4^-/-^* mice (Lu-KO) are used to identify DEGs (differentially expressed genes) in Lu-KO relative to Lu-WT. **(B–D)** Enrichment analysis was performed based on GO cellular component **(B)**, MSigDB hallmark **(C)** and PID **(D)**. **(E)** mRNA levels of TGF-*β negative regulators assayed using qPCR. Data were normalized to Gapdh*. Data is presented as individual data points with mean and 95% confidence intervals. The *p*-values were calculated using *t*-test with Welch’s correction. *p < 0.05.

### 3.4 Pulmonary neutrophils promote a MET-like shift and suppress TGF-*β* signaling in a NET-dependent manner

It has been reported that Smad7 blocks Smad signaling not only by inhibiting Smad phosphorylation but also by interacting with a Smad ubiquitination-related factor (Smurf) 1 and 2 to degrade the type I TGF-*β* receptors and/or Smad proteins (25, 26). To investigate whether TGF-*β* signaling is indeed negatively regulated in the presence of NETs, we examined the expression of TGF-*β* and Smad2/3. The expression levels of both *Tgfβ1* mRNA and Smad2/3 protein were significantly downregulated in Lu-WT cells compared to Lu-KO cells, suggesting that TGF-*β* signaling was attenuated in an autocrine manner within the metastatic microenvironment (**Figures 4A, B**). These findings were supported by the observation that stimulation of Lu-KO cells with SB431542(a TGF-*β* kinase inhibitor led to increased expression of E-cadherin and Ki-67 (**Supplementary Figure 3**). To directly evaluate whether pulmonary neutrophils and their capacity to form NETs can instruct the MET-like phenotypic switch in DTCs, we established an in vitro co-culture system using the mesenchymal-like Lu-KO cells. Because neutrophils are well-documented to be actively involved in the formation of the pre-metastatic niche (27), pulmonary neutrophils were isolated from both WT and *Padi4^-^*^/-^ mice on the exact day of primary tumor resection to capture their primed, pathogenically relevant state. Lu-KO cells cultured in the absence of neutrophils served as the negative control. Remarkably, co-culture with WT pulmonary neutrophils robustly induced the upregulation of epithelial markers, such as E-cadherin and EpCAM, in Lu-KO cells compared to the control groups (**Figures 4C–E**). Additionally, the proportion of Ki-67-positive proliferating cells was significantly increased following co-culture with WT neutrophils (**Figures 4D, E**). Conversely, these epithelial shifts and proliferative gains were largely diminished when Lu-KO cells were co-cultured with *Padi4^-/^*^-^ neutrophils. To further determine whether the physical NET scaffold is functionally required for this phenotypic transition, Lu-KO cells were co-cultured with WT pulmonary neutrophils in the presence or absence of DNase I. Enzymatic degradation of NET scaffolds markedly attenuated the neutrophil-induced restoration of E-cadherin expression and significantly reduced Ki-67 positive cells in Lu-KO cells (**Supplementary Figure 4**). We confirmed that neutrophils isolated from WT mice (purity ≥ 96%) exhibited higher CD11b expression than those isolated from *Padi4^-/-^* mice. Therefore, we inferred that wild-type neutrophils are in a more activated state (**Supplementary Figure 5**). Furthermore, quantitative PCR analysis revealed that co-culture with WT neutrophils, but not *Padi4^-/-^* neutrophils, significantly upregulated the mRNA expression levels of key TGF-*β* negative regulators (**Figure 4F**). Collectively, these findings demonstrate that interaction with NET-competent pulmonary neutrophils suppresses TGF-*β* signaling via the induction of these negative regulators, thereby facilitating a MET-like phenotype shift. Moreover, the sensitivity of this process to DNase I treatment confirms that the physical NET structure plays an essential role, likely acting in concert with neutrophil-derived microenvironmental cues to support the proliferative reactivation of disseminated TNBC cells.

**Figure 4:**
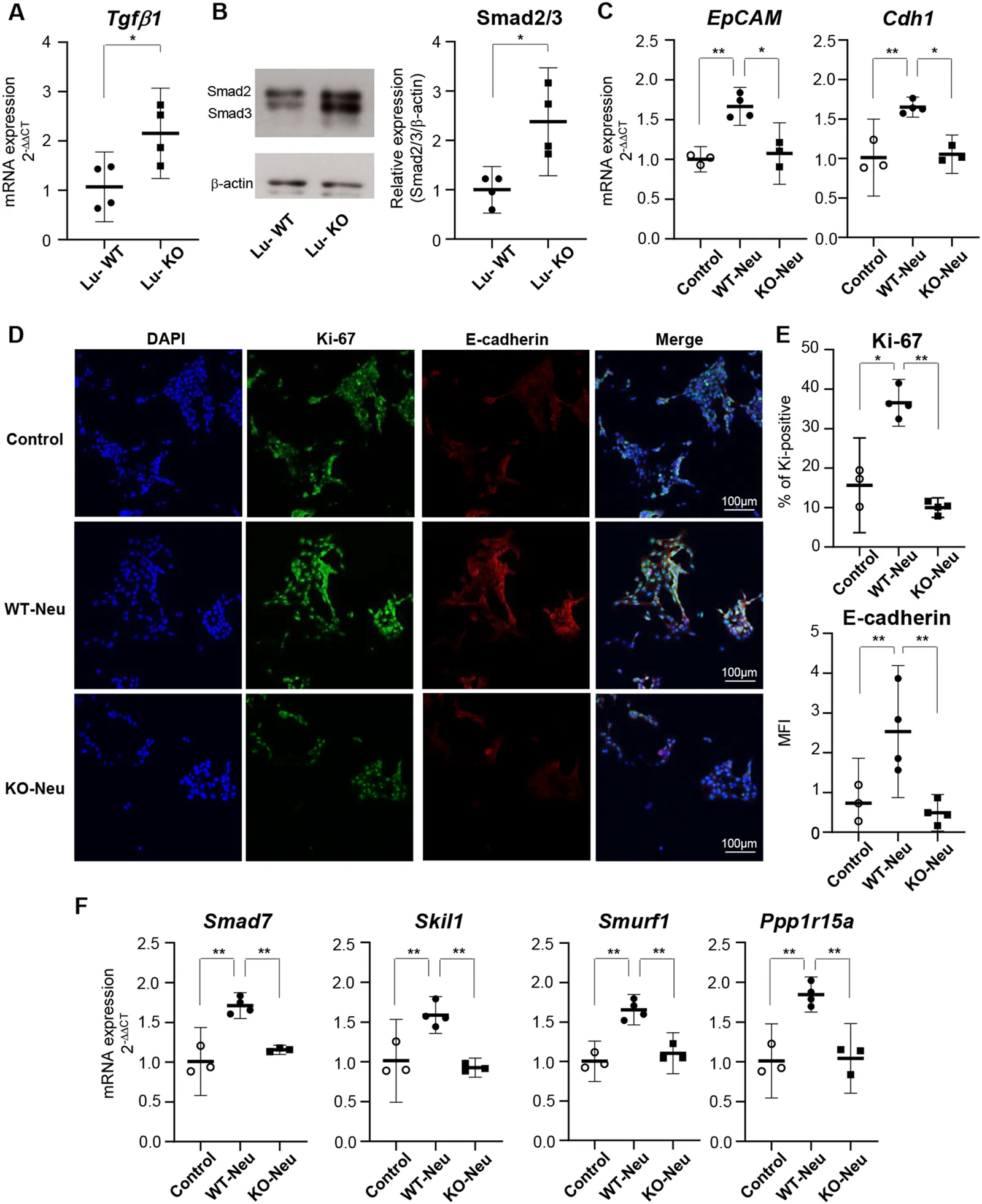
Co-culture with neutrophils promotes epithelial shifts. **(A)** mRNA level of Tgf*β1* assayed using quantitative RT-PCR. Data were normalized to Gapdh. **(B)** Representative plot of Smad2/3 in Lu-WT and Lu-KO (left) and relative band intensity normalized to those of β-actin (right) of four individual samples are shown as mean ± SEM. **(C–E)** Lu-KO cells were co-cultured with either neutrophils from lung of WT mice (n = 4) or those of *Padi4^-/-^* mice (n = 3 or 4) at 1:1 ratio for 48 hours. Lu-KO cells that has not been stimulated by neutrophils were used as controls. **(C, F)** mRNA transcript levels of indicated markers in control, WT-neu and KO-neu were assessed upon quantitative RT-PCR. Data were normalized to *Gapdh*. **(D)** Representative immunofluorescence images of Ki-67staining (green) and E-cadherin (left). **(E)** Percent of Ki-67 positive cells (upper) and mean fluorescence intensity (MFI) of E-cadherin expression (lower) were calculated using ImageJ software. Data is presented as individual data points with mean and 95% confidence intervals. The *p*-values were calculated using *t-*test with Welch’s correction or one-way ANOVA followed by Tukey’s multiple comparison test. *p < 0.05. **p < 0.01.

## 4 Discussion

In this study, we demonstrated that neutrophil extracellular traps (NETs) promote the pulmonary colonization and outgrowth of TNBC by driving a MET-like transition. Using an acute recurrent metastasis model, we showed that *Padi4^-/-^* significantly restricts lung metastasis and prolongs survival after primary tumor resection. Mechanistically, NETs within the lung microenvironment suppress autocrine TGF-*β* signaling in DTCs by upregulating key regulators, including *Smad7* and *Smurf1*. This antagonism triggers a phenotypic switch that reactivates the proliferative capacity of tumor cells. Overall, our findings reveal that NETs act as instructive signals orchestrating tumor cell plasticity, in addition to their established role as physical scaffolds in the metastatic niche (11).

While the awakening of disseminated tumor cells from prolonged dormancy is frequently driven by exogenous inflammatory factors such as infections (28) or smoking and/or LPS injection (29), the clinical reality of TNBC -characterized by a short latency period and acute recurrence- suggests a fundamentally distinct process. In our acute recurrent metastasis model, the pivotal event is more accurately described as a rapid escape from transient cellular quiescence rather than a slow arousal from deeply established, long-term dormancy. Crucially, the tumor-induced inflammatory microenvironment (30), specifically the localized induction of NETs, serves as a definitive microenvironmental cue that favors this exit from quiescence. This autonomous inflammation-NETs axis may explain how TNBC achieves its characteristic rapid metastatic outgrowth and provides a molecular rationale for the timing of clinical relapses.

Several studies previously reported that purified NETs from blood neutrophils promoted EMT and enhanced tumor aggressiveness in various cancers (31–33). Intriguingly, however, our preliminary data indicated that treatment with purified NETs from bone marrow neutrophils alone failed to induce the MET-like transition observed in our models (data not shown). This discrepancy strongly suggests that the capacity of NETs to modulate tumor cell plasticity toward an epithelial phenotype is not merely a consequence of isolated baseline components, such as cell-free DNA or specific proteases acting in a vacuum. Rather, it indicates that the functional output of NETs highly depends on the specific context of the lung microenvironment, where, for instance, pulmonary mesenchymal stromal cells are known to induce NETs via complement C3 expression in response to Th2 cytokines (34). We hypothesize that adaptation to the lung inflammatory environment and localized “education” of neutrophils within this pulmonary niche is a critical process that alters their cell-surface or NET-bound molecular profiles. Through such site-specific reprogramming, tissue-adapted NETs may function as concentrated signaling platforms capable of simultaneously presenting multiple ligands and capturing locally secreted lung-derived factors. We noted that WT pulmonary neutrophils exhibited higher CD11b expression than *Padi4^-/-^* neutrophils, indicating a more activated state. This difference may stem from cell-intrinsic Padi4 signaling or a missing NET-dependent positive feedback loop in *Padi4^-/-^* mice. While explicit MET-inducing cytokines from neutrophils remain uncharacterized, we cannot exclude potential confounding effects of other neutrophil -derived soluble factors, such as IL-1β or TNF-α. These soluble factors likely act synergistically with physical NET scaffold: this complex structure enables cooperative and multivalent interactions with tumor cells, providing the specific co-stimulatory signals necessary to activate the downstream NF-κB pathway and subsequently induce upregulation of TGF-*β* negative regulators (**Figure 3C**). Indeed, as shown in **Figure 4**, co-culture with neutrophils purified from wild-type lungs has been shown to induce a shift toward the epithelial lineage. Although the precise details of the intermolecular interactions remain unclear, our data emphasize that the ability of NETs to induce this shift is not merely a property of the NETs components, but rather a novel property arising from cellular adaptation to the pulmonary microenvironment.

In conclusion, our study demonstrates that, in addition to their physical role, NETs serve a new function as a cue that regulates the plasticity of tumor cells. We demonstrated that tissue-adapted neutrophils function as molecular-level triggers that promote the exit from cellular dormancy. Clinically, this suggests that the perioperative period represents a critical therapeutic window, and that targeting NET formation via PADI4 inhibition treatment may effectively prevent the reactivation of proliferating disseminated tumor cells.

## Data Availability

The datasets presented in this study can be found in online repositories. The names of the repository/repositories and accession number(s) can be found below: https://www.ncbi.nlm.nih.gov/geo/, GSE333273.
