# Supplementary material for: Neutrophil extracellular traps promote lung colonization of triple-negative breast cancer by inducing mesenchymal to epithelial transition
Source: Front Immunol. 2026 Sep 16;17:1892317. doi: 10.3389/fimmu.2026.1892317 (PMC13623897; doi:10.3389/fimmu.2026.1892317)
Supplement: Supplementary file 1 [file DataSheet1.docx]

# Supplementary Figure 1

**Supplementary Figure 1. The compositions of infiltrating neutrophils, macrophages and mMDSCs in primary tumors.**

(A) Representative plots of infiltrating neutrophils (Neu), macrophages (Mac) and mMDSCs in 4T1 primary tumors. (B) Percentage of indicated population within CD45^+^ cells. Data are presented as individual points with mean and 95% confidence intervals.

# Supplementary Figures 2

## Supplementary Figure 2. Immunofluorescence detection of citrullinated H3 (Cit-H3) expression in the WT metastasis lungs.

Representative immunofluorescence images of lung infiltrating neutrophils (Ly6G, green) and NETs (Cit-H3, red). Similar results were obtained from lungs of more than 3 mice per group.

# Supplementary Figures 3

**Supplementary Figure 3. Blockade of TGF-*β* signaling promotes MET-like shift.**

(A) Representative immunofluorescence images of Ki-67 staining (green) and E-cadherin (red).　Lu-KO cells were stimulated with 10μM SB431542 for 24 hours. (B) Percent of Ki-67 positive cells (left) and mean fluorescence intensity (MFI) of E-cadherin expression (right) were calculated using ImageJ software. Data are presented as individual data points with mean and 95% confidence intervals. The *p*-values were calculated using *t-*test with Welch’s correction. **p* < 0.05.

**Supplementary Figures 4**

**Supplementary Figure 4. DNase I treatment attenuates the MET-like phenotypic shift in co-culture.**

(A) Representative immunofluorescence images of Ki-67 staining (green) and E-cadherin (red) in Lu-KO cells co-cultured with WT pulmonary neutrophils in the presence or absence of DNase I (1.5 U/ml). (B) Percentage of Ki-67 positive cells (upper) and mean fluorescence intensity (MFI) of E-cadherin expression (lower) were calculated using ImageJ software. Data are presented as individual data points with mean and 95% confidence intervals. The *p*-values were calculated using one-way ANOVA followed by Tukey’s multiple comparison test. ***p* < 0.01

# Supplementary Figures 5

**Supplementary Figure 5. The activation status of neutrophils in pre-metastatic lungs.**

(A) Representative plots of infiltrating neutrophils in the pre-metastatic lungs. (B) CD11b expression on neutrophils from lung of WT mice (n = 4) and those of *Padi4-/-* mice (n = 4) was determined by flow cytometry. Representative histograms with CD11b staining (left) from WT mice in black and *Padi4-/-* mice in gray. Quantification of CD11b expression on neutrophils (right) is presented as the mean fluorescence intensity (MFI). Data are presented as individual data points with mean and 95% confidence intervals. The *p*-values were calculated using *t-*test with Welch’s correction.

**Supplementary Table. Primer sequences for qPCR analysis.**

| Gene | Forward primer | Reverse primer |
| --- | --- | --- |
| *GAPDH* | TGTGTCCGTCGTGGATCTGA | CCTGCTTCACCACCTTCTTGA |
| *EpCAM* | TTTGCTCCAAACTGGCGTCT | TTGTTCTGGATCGCCCCTTC |
| *Cdh1* | AACCCAAGCACGTATCAGGG | GAGTGTTGGGGGCATCATC |
| *Vim* | TCCAGAGAGAGGAAGCCGAA | AAGGTCAAGACGTGCCAGAG |
| *CD51* | TCCGACGAGCACTGTTTCTC | CATCCCGAAGATAGGCGACC |
| *CD106* | TCCTGTGGAGCAGACAGCTA | AAATGCCGGAATCGTCCCTT |
| *Tgfb1* | CTCCCGTGGCTTCTAGTGC | GCCTTAGTTTGGACAGGATCTG |
| *Smad7* | AACCCCCATCACCTTAGTCG | ACAGCCTGCAGTTGGTTTGA |
| *Smurf1* | GACGGCGGGCACAGATTAG | TTTTCCATGGCCACACTGGT |
| *Skil1* | AAGGCTCTGCAAGGCTCTAC | CCTTGGGCAGGTTGTCTGTA |
| *Ppp1r15a* | CCCGCCGCCCAGACA | TAGAAGTTGTGGGCGTCCCT |
